# Comparison of Nissen vs. Toupet fundoplication in laparoscopic hiatal hernia repair for gastroesophageal reflux disease with extra-esophageal symptoms

**DOI:** 10.3389/fmedt.2025.1678192

**Published:** 2026-01-09

**Authors:** Qingchao Zhu, Nengquan Sheng, Zhigang Wang, Yang Xia

**Affiliations:** Department of Surgery, Shanghai Jiao Tong University Affiliated Sixth People’s Hospital, Shanghai, China

**Keywords:** gastroesophageal reflux disease (GERD), hiatal hernia (HH), laparoscopic hiatal hernia repair (LHHR), anti-reflux, EORTC QLQ-C30

## Abstract

**Objective:**

This study aims to evaluate the clinical efficacy of laparoscopic hiatal hernia repair (LHHR) in treating gastroesophageal reflux disease (GERD) and to through the therapeutic effect of total (360°) and partial (270°) laparoscopic fundoplication.

**Methods:**

This retrospective observational study enrolled 100 patients, both with and without documented extra-oesophageal symptoms of GERD. Data were extracted from medical records, covering basic information, symptoms, treatments, and follow-up. Symptom relief and quality of life were assessed via GERD-Q score, Reflux Symptom Index (RSI), and EORTC QLQ-C30 scale, offering a foundation for comprehensive GERD patient management and treatment evaluation in clinical practice.

**Results:**

The DeMeester index significantly decreased postoperatively in both the laparoscopic Nissen fundoplication (LNF) group (from 55.23 ± 25.12 to 11.45 ± 10.20, *p* < 0.05) and the laparoscopic Toupet fundoplication (LTF) group (from 60.51 ± 28.40 to 11.70 ± 9.65, *p* < 0.05). The RSI scores improved at 12 months postoperatively in both groups: LNF group (from 23.1 ± 15.4 to 13.7 ± 9.6, *p* < 0.05) and LTF group (from 21.9 ± 15.8 to 12.8 ± 8.2, *p* < 0.05). The GERD scores also improved postoperatively: LNF group (from 13 ± 5.0 to 10 ± 4.4, *p* < 0.05) and LTF group (from 10 ± 4.7 to 7.5 ± 4.5, *p* < 0.05).

**Conclusion:**

Our report demonstrates that LHHR significantly improved GERD regarding symptom frequency, acid reflux occurrences and DeMeester score. Both LNF and LTF provide good results.

## 1 Introduction

Gastroesophageal reflux disease (GERD) is a common digestive system disease, which is characterized by reflux of gastric acid or other gastric contents into the esophagus, causing a series of symptoms, such as heartburn, acid regurgitation, dysphagia, etc. (1, 2). The most recognizable symptoms of GERD are acid reflux and heartburn (3). GERD with extra-esophageal (EE) symptoms (GERD-EE) is a subset of GERD that leads to laryngitis (10.4%), cough (13%), dysphonia (14.8%), asthma (9.3%), pulmonary fibrosis and pneumonia (4–6). The disease not only affects the quality of life of patients but also can lead to complications such as esophagitis and esophageal stenosis.

Hiatal hernia (HH), a common anatomical defect associated with GERD, is managed through GERD medications that control symptoms rather than correct reflux pathophysiology (7). While proton-pump inhibitors (PPIs) effectively relieve typical GERD symptoms, their efficacy diminishes significantly for extra-esophageal manifestations (8). Poor response to medication should alert us to potential anatomical abnormalities such as hiatal hernia of the esophagus, which can be further evaluated by imaging or endoscopy. For GERD patients with combined paraoesophageal hernia, hernia sac retraction needs to be accomplished as a priority and whereas GERD repair of simple sliding hernia is performed with fundoplication as the core operative gastric step (9). Laparoscopic hiatal hernia repair (LHHR) has become the valid intervention for symptomatic cases, demonstrating lower perioperative morbidity and shorter hospitalization compared to open procedures (10). At present, robotic surgical solutions have been developed, compare the LHHR with robotic HHR, the perioperative complication rate is similar and the robotic HHR cost is higher (11). Mesh as a common surgical material in HHR, in the surgical outcome, the type of mesh has no significance influential difference except the cost (12).

Surgical treatment of GERD involves laparoscopic fundoplication, where the gastric fundus is wrapped around the distal esophagus to restore the antireflux barrier (13). The two predominant techniques are laparoscopic Nissen (LNF) and Toupet fundoplication (LTF), though multiple variants exist (including Nissen, Toupet, Dor, and Watson techniques) without consensus on optimal approach (14). While meta-analyses have compared LNF and LTF outcomes for typical symptoms (15, 16), evidence remains limited regarding their impact on extra-esophageal manifestations.

In this study, we aimed to evaluate the clinical efficacy of LHHR in treating GERD and to compare the therapeutic effect of LNF and LTF. The postoperative improvements of LHHR were evaluated on these patients in terms of the GERD-Q Symptom Scale, 24 h pH monitoring of the esophagus, high-resolution esophageal manometry, and barium upper gastrointestinal examination.

## 2 Methods

### 2.1 Design & patients

This retrospective observational study included 100 patients with or without documented extra-esophageal GERD symptoms between January 2021 to December 2023. The study was approved by the Medical Ethics Committee of our hospital [2021-KY-111(K)]. Written informed consent was obtained from all participants before their enrollment in the study.

### 2.2 Data collection

**Figure 1:**
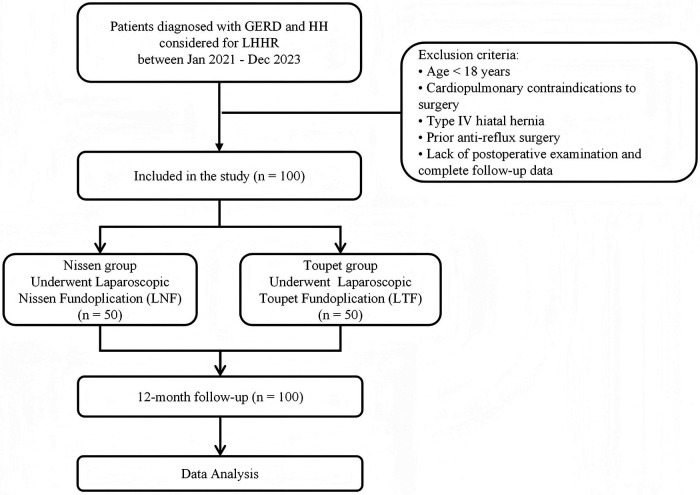
Patient enrollment and study flow diagram.

Data were collected retrospectively by reviewing the patients' medical records. Baseline characteristics were collected such as general patient's condition (age, gender, BMI, etc.) and GERD characteristics (type of HH, comorbidities, DeMeester score before procedure, details of sub-scores and treatment history, esophageal and extra-esophageal symptoms, etc.) (Figure 1). Per preoperative and postoperative characteristics were collected as well.

### 2.3 Inclusion criteria

Diagnosis involved a comprehensive preoperative workup for all patients, which included clinical evaluation of esophageal/extra-esophageal symptoms, endoscopic confirmation of esophagitis, 24 h pH monitoring to quantify acid exposure, high-resolution esophageal manometry, and barium swallow/endoscopy for hiatal hernia detection (17, 18). All patients received anti-reflux medications and multidisciplinary otolaryngological/respiratory evaluations. High-resolution manometry was performed routinely to assess esophageal motility. For patients with severe dysphagia or odynophagia to exclude major motility disorders that could influence the choice of fundoplication type.

### 2.4 Exclusion criteria

Patients age under 18 years; Patients with cardiopulmonary diseases that cannot tolerate surgery; Patients with a combination of giant esophageal HH (type IV), who intend to undergo other surgeries or who have had anti-reflux surgery in the past; Patients without relevant postoperative examination and follow-up.

### 2.5 Preoperative workup

#### 2.5.1 High-resolution esophageal manometry

All enrolled patients underwent high-resolution esophageal manometry preoperatively and postoperatively. This system has 12 pressure sensing points around each channel to detect and measure the length of the LES, thoracic and abdominal esophageal pressures, respiratory changes, and pressure changes in each index. Pressure measurements at the esophagogastric junction (EGJ) can be used to assist in the assessment of the function of the regurgitant barrier, but needs to be combined with endoscopy, dynamic pH monitoring and other comprehensive assessments of GERD. It is anti-reflux when the diaphragmatic pedicle is normally contracted and is pathologically hypo-refluxive and can be used for preoperative evaluation of esophageal HH repair and anti-reflux surgery. On examination, if the EGJ forms two separate high-pressure bands, the upper band indicates LESP, while the lower band suggests that the esophageal hiatus may be compressing the gastric fundus tissue, indicating the presence of HH. The longer the length, the worse the outcome of drug therapy may be. The reference standard in this study was the lower esophageal sphincter pressure (LESP), and its change value before and after surgery to assess esophageal pressure recovery and evaluate the surgical effect.

#### 2.5.2 24-hour postoperative esophageal pH acidity measurements

Enrolled patients underwent preoperative and 24 h postoperative esophageal pH acidity measurements. During the examination period, patients are asked to stop using drugs that inhibit gastric acid secretion and affect gastrointestinal motility for at least one week. During this period, patients should avoid consuming raw, cold, and spicy foods. On the morning of the test, the patient woke up on an empty stomach and placed the acid electrode catheter into the esophagus through the nostril. It is then deepened until the pH value on the indicator box suddenly drops below 4, i.e., into the gastric cavity. The electrode catheter is then slowly retracted using the gradient method until the pH value shows between 5.5 and 7, indicating a position approximately near the LES. While the depth can be recorded and compared with the depth of esophageal manometry to determine if the acid-conducting catheter is broken. The catheter is retracted approximately 5 cm to secure the catheter, and the sensing device is worn and recorded for 24 h. Simultaneous pharyngeal pH measurements are performed with the electrode catheter placed and suspended approximately 0.5 cm below the esophageal catheter level, following a procedure essentially identical to esophageal acid measurement.

#### 2.5.3 Barium radiology

Radiography was carried out by an experienced radiologist. The RE diagnosis was made according to Christiansen' s method. HH was diagnosed if the gastric folds extended above the hiatus. If the diaphragmatic esophageal tear hole is found in the development, or the gastric mucosa morphology entering the thoracic cavity can be found directly, the diagnosis can be clearly made. This examination is meaningful for understanding the food propulsion function and clearance ability of the esophagus, the morphology of the stomach, mucous membrane and peristalsis, and is also valuable for the location, size and classification of HH.

### 2.6 Surgical technique

LHHR: Patients received general anesthesia, an indwelling gastric tube and routine 5-well method (5 trocar needles) procedure. Briefly, the herniated gastric tissue was pulled back into the abdominal cavity. The abdominal esophagus and the stomach fundus and the posterior esophagus space were exposed. The stomach fundus was pulled to the lower right to separate the spleen, and the diaphragmatic esophageal membrane was opened. The hernia sac wall was removed, and 3-0 absorption barbed suture was used to continuously suture esophageal hole with 4-0 non-absorption suture reinforcement. A mesh was used for large esophageal holes (5–6 cm^2^).

LTF or LNF: The selection of fundoplication type was guided by preoperative manometry, with LNF performed for a hypotensive lower esophageal sphincter and LTF for preserved LES pressure or esophageal dysmotility. All laparoscopic anti-reflux procedures were performed by a single surgeon following standard techniques: hiatal closure with bilateral crural dissection, full fundus mobilization with short gastric vessel division, and creating ≤2 cm 360° wraps (Group I) or 270° fundoplications (Group II) using a 56-Fr bougie. Key considerations included maintaining ≥3 cm intra-abdominal esophagus and incorporating vagus nerves in the wrap. A 360-degree Nissen and a 270-degree Toupet fundoplication were performed in Supplementary Figures S1, S2.

During postoperative 10 days, all patients received a semi-liquid diet with gradual transition to solid foods under medical supervision to prevent dysphagia.

### 2.7 Postoperative assessment

The same gastrointestinal surgeon performed clinical assessments preoperatively and at 12-month follow-up. Data were collected via structured clinical interviews, supplemented by telephone interviews and standardized questionnaires as needed. confirmed the patients restored through a combination of the GERD-Q Symptom Scale, 24 h pH monitoring of the esophagus, high-resolution esophageal manometry, and barium upper gastrointestinal examination. If there are no relevant surgical complications, they should return to the hospital regularly after discharge and follow-up the postoperative quality of life by the EORTC QLQ-C30 Evaluation Scale. At least 3 days prior to clinical evaluation, acid-suppressing medications were discontinued.

### 2.8 The GERD-Q, RSI and EORTC QLQ-C30

1. Basic patient information, including gender, age, height, weight, and occupation, was collected, and the GERD-Q (19) was administered to record symptoms experienced over the past 7 days.
  (A) The frequency of positive symptoms (heartburn, reflux) was scored as 0, 1, 2, 3, and 6 points for 0, 1, 2–3, and 4–7 days, respectively;
  (B) The frequency of symptoms negatively associated with GERD (epigastric pain, nausea) was scored as 3, 2, 1, and 0 points for days 0, 1, 2–3, and 4–7, respectively.
  (C) The frequency of positive symptoms affecting nocturnal sleep (heartburn, reflux) and the frequency of additional medication were scored as 0, 1, 2, and 3 points for 0, 1, 2–3, and 4–7 days, respectively.

The GERD-Q score for each patient is the cumulative sum of the scores for the three symptom categories mentioned above.

2. Individuals with extra-esophageal reflux symptoms were assessed using the RSI (20), which includes nine symptoms: hoarseness or dysphonia, persistent throat clearing, excessive sputum or nasal discharge, difficulty swallowing food, water, or tablets, coughing after a meal or while lying down, dyspnea, a foreign-body sensation in the throat, bothersome cough, heartburn, chest pain, and stomach pain. Each symptom was rated on a 6-point scale from “asymptomatic” to “very severe” (0–5). A total score of RSI >13 is considered abnormal.
3. After LHHR of the oesophagus, patients should be discharged from the hospital and returned regularly for review and assessment of postoperative quality of life if recovery is uneventful and there are no associated surgical complications. The EORTC QLQ-C30 (21) evaluation scale was used to evaluate the functional status and general health of the patients in each group 1 year after surgery. Postoperative quality of life was assessed and compared. In the questionnaire, 0 means never; 1 means rarely; 2 means sometimes; 3 means often; and 4 means frequently. The EORTC QLQ-C30 evaluation scale was used to assess the patients' quality of life. This 11-item questionnaire was designed to measure the effect of LHHR on functional scales (bodily-functional, role-functional, affective-functional, cognitive-functional, social-functional) and symptomatic scales (heartburn, dysphagia, reflux, hiccups, abdominal distension, flatulence). The EORTC QLQ-C30 Evaluation Scale reflects the daily psychological and physiological status. Patients were asked to record their daily physical condition, with lower scores indicating that postoperative sequelae had less impact.

## 3 Statistical analysis

The DeMeester score was included as a continuous variable for analysis. Additionally, a score >14.7 was used to define the presence of pathological gastroesophageal reflux.

All statistical analyses were performed using SPSS 26.0, with the significance level set at *α* = 0.05. Continuous variables, confirmed to be normally distributed by Kolmogorov–Smirnov and Shapiro–Wilk tests, are expressed as mean ± standard deviation (x¯ ± s). Comparisons between two groups were conducted using the independent samples *t*-test. Categorical variables are presented as frequencies (percentages) and compared using Fisher's exact test or Chi-square test, as appropriate.

## 4 Result

This project documented the outcomes before and after LNF or LTF surgery for the treatment of GERD. The degree of symptomatic relief and the evaluation of relevant objective indicators in patients who underwent surgical treatment were observed. LHHR, including LTF and LNF, significantly improved GERD in respect of symptom frequency, number of acid reflux occurrences and DeMeester score. LNF and LTF demonstrate equivalent efficacy in treating GERD patients with extra-esophageal manifestations.

One hundred patients with documented GERD and extra-esophageal reflux symptoms were enrolled in this study. In the LTF group, the patient demographic consisted of 12 females and 38 males, summing up to a total of 50 patients. Similarly, the LNF group comprised 10 females and 40 males, also totaling 50 patients. The average age of the patients in the LNF group was 52.60 ± 7.90 years, while in the LTF group, it was 54.65 ± 8.35 years. Leading comorbidities among the study population were hypertension (LTF = 44.0%, LNF = 36%), hyperlipidemia (LTF = 36.0%, LNF = 30.0%), arrhythmia (LTF = 6.0%, LNF = 12.0%), coronary artery disease (LTF = 6.0%, LNF = 6.0%), and diabetes mellitus (LTF = 4.0%, LNF = 4.0%). Demographic data showed no significant differences between LNF and LTF groups. Typical GERD symptoms affected >50% of patients (heartburn 56.0%, regurgitation 48.0%). Predominant atypical manifestations included cough (71.0%), globus pharyngeus (63.0%), and throat clearing (52.0%).

Both groups underwent successful laparoscopic procedures without open conversion. No major complications or mortality occurred, with all patients receiving PPI therapy. Patient characteristics and comparative outcomes between LNF and LTF groups are summarized in Tables 1, 2.

**Table 1:** Patient basic information (*n* = 100).

Characteristic	Nissen group (*n* = 50)	Toupet group (*n* = 50)	*P* value
Age, mean ± SD, years	52.60 ± 7.90	54.65 ± 8.35	0.210
Male, *n* (%)	38 (76.0)	40 (80.0)	0.718
Female, *n* (%)	12 (24.0)	10 (20.0)	0.720
BMI, mean ± SD, kg/m^2^	20.65 ± 3.12	21.79 ± 2.85	0.059
Years with GERD, mean ± SD	4.1 ± 1.8	4.3 ± 1.3	0.525
Years with PPI use, mean ± SD	3.4 ± 2.2	3.3 ± 1.9	0.811
Type of HH			
Ⅰ, *n* (%)	35 (70.0)	29 (58.0)	0.453
Ⅱ, *n* (%)	3 (6.0)	1 (2.0)	0.317
Ⅲ, *n* (%)	12 (24.0)	20 (40.0)	0.157
Comorbidities			
Smoking, *n* (%)	15 (30.0)	25 (50.0)	0.228
Hypertension, *n* (%)	14 (28.0)	22 (44.0)	0.365
Hyperlipidemia, *n* (%)	13 (26.0)	18 (36.0)	0.739
Arrhythmia, *n* (%)	6 (12.0)	3 (6.0)	0.635
Coronary artery disease, *n* (%)	3 (6.0)	3 (6.0)	1.000
History of myocardial infarction, *n* (%)	1 (2.0)	0 (0.0)	0.635
Diabetes mellitus, *n* (%)	2 (4.0)	2 (4.0)	1.000
Chronic renal disease, *n* (%)	0 (0.0)	1 (2.0)	0.635

**Table 2:** Preoperative GERD symptoms and esophageal 24 h pH acidity measurement data.

Characteristic	Nissen group (*n* = 50)	Toupet group (*n* = 50)	*P* value
Total number of regurgitations	62.32 ± 24.65	64.45 ± 25.84	0.676
Number of acid refluxes	19.89 ± 3.29	21.01 ± 3.77	0.116
Weakly acidic reflux	25.82 ± 4.09	27.21 ± 4.58	0.111
Non-acid countercurrent order	19.82 ± 4.32	19.23 ± 3.25	0.444
DeMeester Score	55.23 ± 25.12	60.51 ± 28.40	0.329
Total time pH < 4, min	298.40 ± 94.80	310.10 ± 68.45	0.479
% time pH < 4, min	23.50 ± 5.90	25.31 ± 7.30	0.175
% time upright pH < 4.0	7.58 ± 3.98	7.21 ± 3.63	0.627
% time supine pH < 4.0	11.65 ± 3.22	12.24 ± 5.45	0.512
Number lasting 5 min reflux	9.80 ± 6.10	10.30 ± 8.14	0.727
LESP, mmHg, mean ± SD	7.50 ± 5.10	8.58 ± 6.56	0.360
Esophageal symptoms			
Heartburn, *n* (%)	27 (54.0)	29 (58.0)	0.789
Regurgitation, *n* (%)	23 (46.0)	25 (50.0)	0.773
Extra-esophageal symptoms			
Postnasal drip, *n* (%)	10 (20.0)	18 (36.0)	0.131
Hoarseness, *n* (%)	12 (24.0)	13 (26.0)	0.842
Globus pharyngeus, *n* (%)	33 (66.0)	30 (60.0)	0.706
Cough, *n* (%)	38 (76.0)	33 (66.0)	0.553
Throat clearing, *n* (%)	25 (50.0)	27 (54.0)	0.782

**Figure 2:**
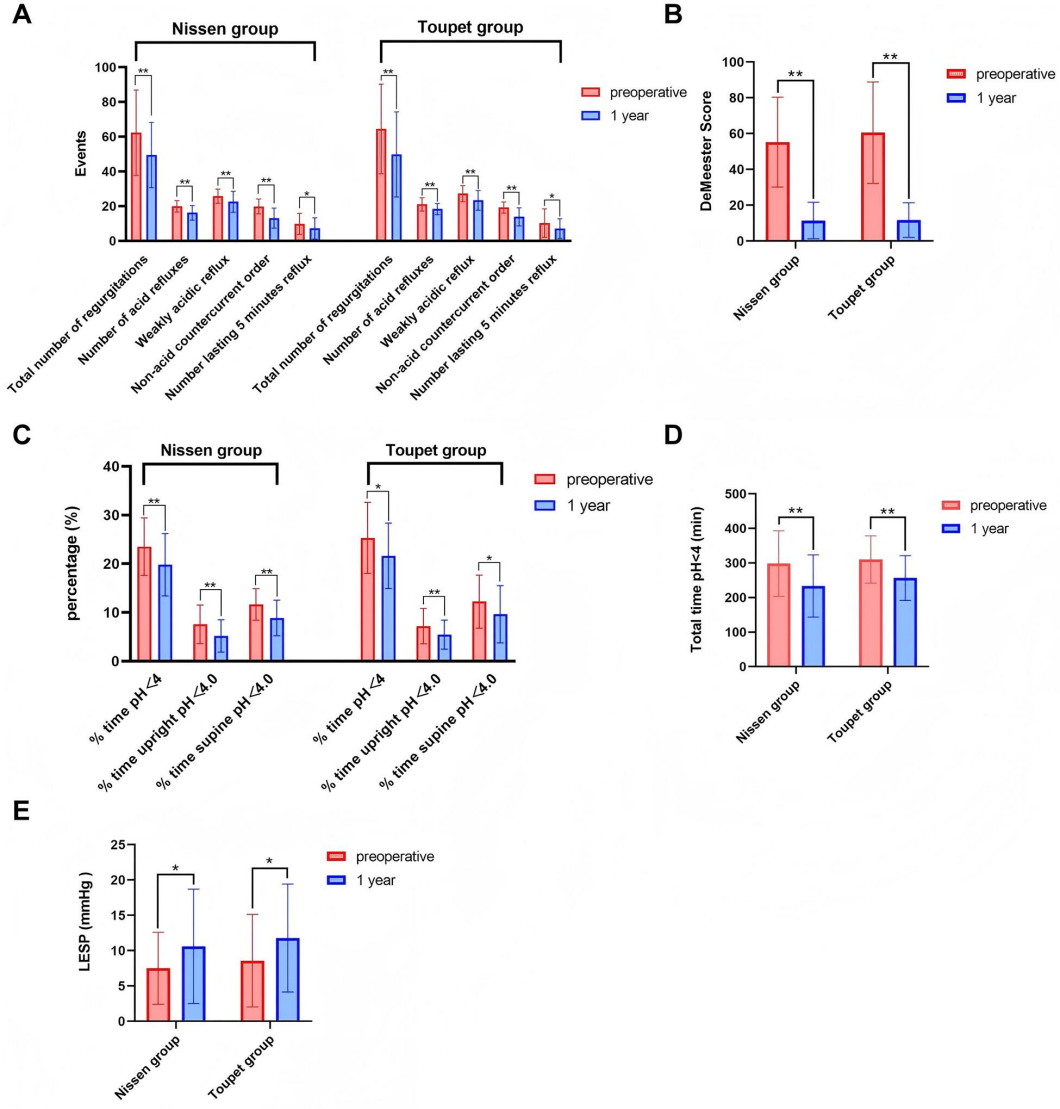
Comparison of preoperative and postoperative data in a single group. **(A)** The typical and atypical symptoms compared between preoperative and postoperative patients in the Nissen and Toupet grouops. **(B)** DeMeester Score compared between preoperative and postoperative patients in the Nissen and Toupet grouops. **(C)** The percentage of time pH < 4, time upright pH < 4.0 and time supine pH < 4.0 compared between preoperative and postoperative patients in the Nissen and Toupet grouops. **(D)** The total time pH < 4 compared between preoperative and postoperative patients in the Nissen and Toupet grouops. **(E)** LESP compared between preoperative and postoperative patients in the Nissen and Toupet grouops. Data are presented as mean ± SD. Statistical significance was determined using the independent samples *t*-test. *: *P* < 0.05, **: *P* < 0.01. LESP, lower esophageal sphincter pressure.

In most patients, a significant regression or complete resolution of preoperative symptoms was achieved. Both typical and atypical symptoms of all patients were significantly improved in both LTF and LNF groups (*P* < 0.05, Table 3). Results of 24h-pH monitoring demonstrated a decreased DeMeester index, from 55.23 ± 25.12 to 11.45 ± 10.20 in LNF and 60.51 ± 28.40 to 11.70 ± 9.65 in LTF (*P* < 0.05). The DeMeester score was close to the normal value, and esophageal inflammation improved, effectively enhancing the quality of life in the prognosis (Table 3, Figure 2).

**Table 3:** Comparison of preoperative and postoperative data in a single group.

Characteristic	Nissen group			Toupet group		
	Preoperative	1 year	*P* value	Preoperative	1 year	*P* value
Total number of regurgitations	62.32 ± 24.65	49.48 ± 18.76	0.004	64.45 ± 25.84	49.80 ± 24.50	0.004
Number of acid refluxes	19.89 ± 3.29	16.18 ± 4.20	0.001	21.01 ± 3.77	18.34 ± 3.21	0.001
Weakly acidic reflux	25.82 ± 4.09	22.52 ± 6.04	0.002	27.21 ± 4.58	23.39 ± 5.68	0.001
Non-acid countercurrent order	19.82 ± 4.32	13.13 ± 5.72	0.001	19.23 ± 3.25	13.86 ± 5.20	0.001
DeMeester Score	55.23 ± 25.12	11.45 ± 10.20	0.001	60.51 ± 28.40	11.70 ± 9.65	0.001
Total time pH < 4, min	298.40 ± 94.80	233.50 ± 90.03	0.001	310.10 ± 68.45	256.76 ± 64.83	0.001
% time pH < 4, min	23.50 ± 5.90	19.80 ± 6.40	0.003	25.31 ± 7.30	21.63 ± 6.72	0.010
% time upright pH < 4.0	7.58 ± 3.98	5.21 ± 3.32	0.002	7.21 ± 3.63	5.46 ± 2.98	0.002
% time supine pH < 4.0	11.65 ± 3.22	8.89 ± 3.65	0.001	12.24 ± 5.45	9.65 ± 5.87	0.023
Number lasting 5 min reflux	9.80 ± 6.10	7.23 ± 6.07	0.036	10.30 ± 8.14	7.02 ± 5.66	0.020
LESP, mmHg, mean ± SD	7.50 ± 5.10	10.60 ± 8.10	0.023	8.58 ± 6.56	11.78 ± 7.65	0.026
Esophageal symptoms						
Heartburn, *n* (%)	27 (54.0)	9 (18.0)	0.003	29 (58.0)	11 (22.0)	0.004
Regurgitation, *n* (%)	23 (46.0)	7 (14.0)	0.004	25 (50.0)	6 (12.0)	0.001
Extra-esophageal symptoms						
Postnasal drip, *n* (%)	10 (20.0)	2 (4.0)	0.042	18 (36.0)	5 (10.0)	0.007
Hoarseness, *n* (%)	12 (24.0)	3 (6.0)	0.020	13 (26.0)	3 (6.0)	0.012
Cough, *n* (%)	38 (76.0)	10 (20.0)	0.001	33 (66.0)	7 (14.0)	0.001
Globus pharyngeus, *n* (%)	33 (66.0)	13 (26.0)	0.003	30 (60.0)	8 (16.0)	0.001
Throat clearing, *n* (%)	25 (50.0)	5 (10.0)	0.001	27 (54.0)	9 (18.0)	0.003

**Figure 3:**
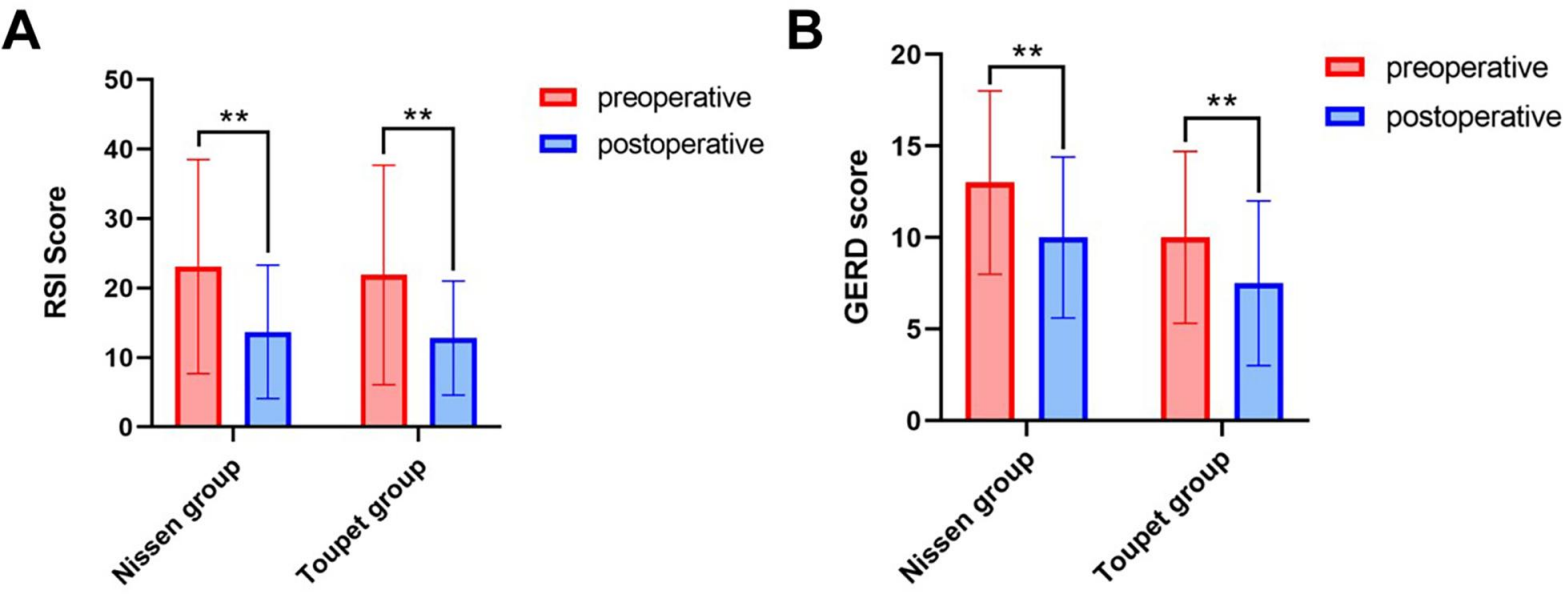
The outcomes of RSI and GERD questionnaire. **(A)** The RSI Score of preoperative and postoperative patients between Nissen and Toupet grouops. **(B)** The GERD Score of preoperative and postoperative patients between Nissen and Toupet grouops. Data are presented as mean ± SD. Statistical significance was determined using the independent samples *t*-test. **: *P* < 0.01. RSI, reflux symptom index; GERD, gastroesophageal reflux disease.

Both surgical groups exhibited significant RSI reductions postoperatively (LNF: 23.1 ± 15.4 to 13.7 ± 9.6; LTF: 21.9 ± 15.8 to 12.8 ± 8.2; both *P* < 0.05). Comparative analysis revealed marked symptom improvement at 12-month follow-up vs. preoperative baselines (Table 4, Figure 3).

**Table 4:** The outcomes of EORTC QLQ-C30 scales, RSI and GERD questionnaire.

Characteristic	Nissen group			Toupet group		
	preoperative	postoperative	*P*-value	Preoperative	postoperative	*P*-value
RSI	23.1 ± 15.4	13.7 ± 9.6	0.001	21.9 ± 15.8	12.8 ± 8.2	0.001
GERD score	13 ± 5.0	10 ± 4.4	0.002	10 ± 4.7	7.5 ± 4.5	0.008
Functional scales			0.034			0.037
Physical functioning	80.61 ± 24.25	91.21 ± 20.89		78.69 ± 18.27	85.98 ± 12.61	
Role functioning	74.68 ± 26.73	88.42 ± 48.12		71.63 ± 21.06	84.21 ± 31.52	
Emotional functioning	55.67 ± 13.77	70.33 ± 45.10		50.61 ± 18.21	68.32 ± 41.27	
Cognitive functioning	74.46 ± 18.64	89.78 ± 14.23		75.79 ± 14.45	87.01 ± 19.84	
Social functioning	74.25 ± 13.68	87.31 ± 20.79		69.62 ± 16.34	85.13 ± 21.64	
Symptom scales			0.025			0.029
Heartburn	37.94 ± 7.16	14.32 ± 6.21		45.36 ± 6.32	18.25 ± 5.28	
Dysphagia	48.57 ± 6.47	19.95 ± 4.32		54.91 ± 4.98	16.84 ± 4.97	
Regurgitate	31.68 ± 5.64	15.32 ± 5.25		38.67 ± 4.60	16.28 ± 7.01	
Burping	56.41 ± 8.65	24.21 ± 4.68		61.24 ± 6.57	26.81 ± 5.69	
Bloatedness	67.58 ± 6.54	29.27 ± 3.25		69.41 ± 5.24	32.59 ± 6.42	
Flatulence	54.65 ± 9.41	29.54 ± 5.21		60.37 ± 8.23	33.92 ± 5.84	

Postoperative analysis revealed significant GERD symptom improvement in both groups. LNF patients showed median score reduction from 13 (SD = 5.0) to 10 (SD = 4.4) at 12 months (*P* < 0.05). Similarly, LTF group scores decreased from 10 (SD = 4.7) to 7.5 (SD = 4.5) (Table 4).

All five areas of functional scores showed significant improvement in patients after surgery. In particular, the physical functioning score was higher in both groups after surgery. While objective tests, including esophageal manometry and 24 h esophageal pH monitoring, performed preoperatively and postoperatively, demonstrated the surgery's effectiveness in reducing the number of pathologic acid reflux episodes, lowering the DeMeester score, and decreasing the severity of GERD. The decreasing total number of refluxes in both LNF group and LTF group demonstrated the surgery's effectiveness in reducing reflux episodes. Additionally, postoperative GERD-Q and RSI scores were significantly reduced (*P* < 0.05), indicating significant improvement in both reflux and associated extra-esophageal symptoms. Patients in both groups showed varying degrees of improvement in physical functioning, role functioning, and emotional functioning, indicating that patients recovered well in daily life and social activities after surgery. Overall, postoperative patients' functional scores improved, and symptom scores decreased, indicating that the surgery effectively improved patients' quality of life and health (Table 4).

**Figure 4:**
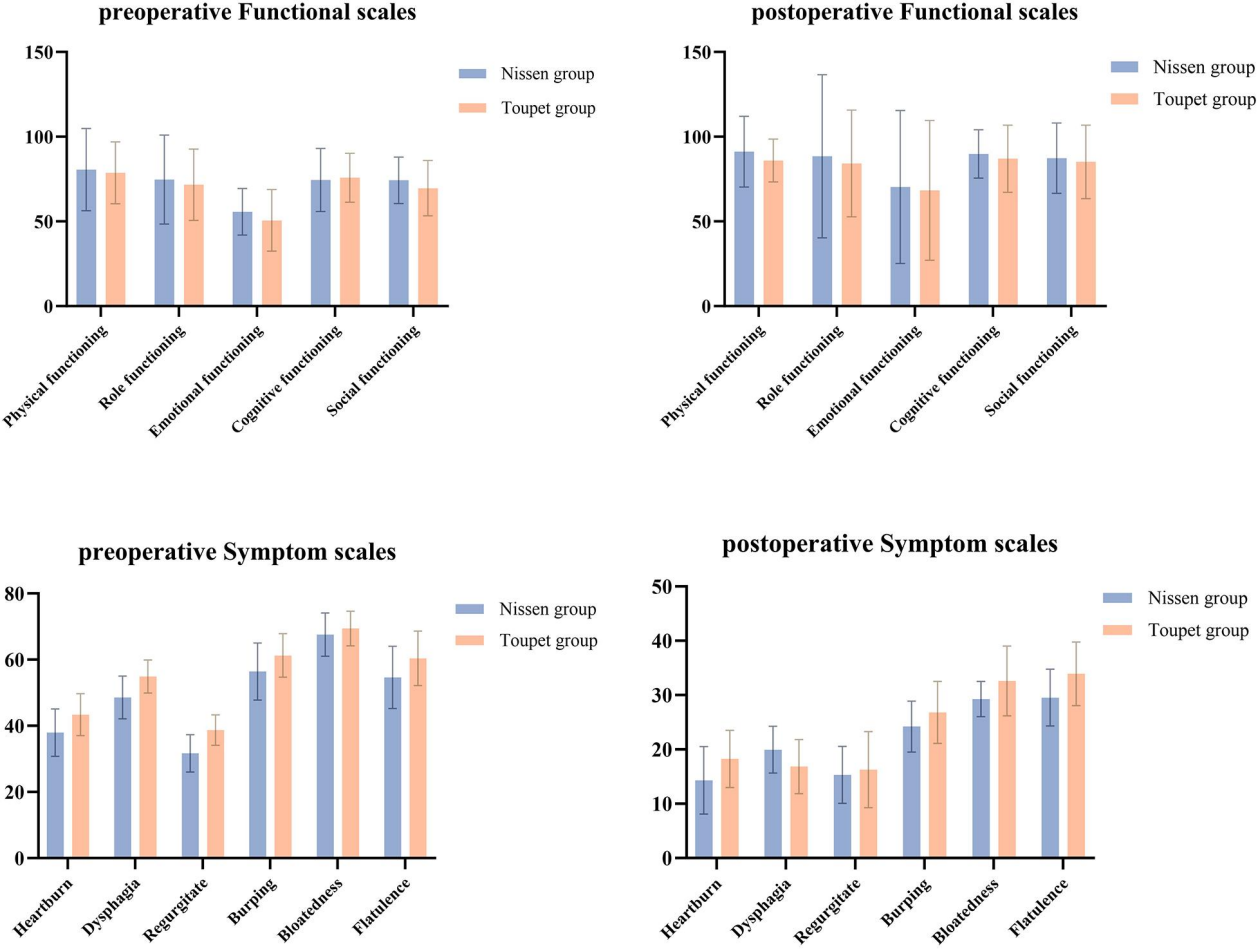
The outcomes of EORTC QLQ-C30 scales.

As assessed by the EORTC QLQ-C30 scale, the follow-up data 12 months after the surgery showed that the patients' quality of life, including physical, emotional, and social functions, improved to different degrees in various indicators (Table 4, Figure 4).

## 5 Discussion

Understanding the complexities of HH and GERD is essential for developing effective treatment strategies. HH, characterized by the displacement of the stomach through the diaphragm, is often associated with GERD, a condition where stomach acid frequently flows back into the esophagus, leading to symptoms such as heartburn and regurgitation. These conditions can significantly impact patients' quality of life and are linked to various comorbidities, making their management a crucial area of clinical research. The relationship between HH and GERD is particularly pertinent, as surgical interventions have shown promise in alleviating symptoms and improving overall patient outcomes. Recent studies indicate that patients with HH associated with GERD may experience varying degrees of symptom severity, which necessitates tailored approaches to treatment and management (22, 23).

In this study, we have made significant strides in understanding the management of HH and GERD, particularly in the context of surgical interventions. Our findings revealed that the acid reflux rate was significantly reduced by LHHR in both groups, suggesting an improvement in the GERD alleviation. Previous studies have primarily focused on either surgical or non-surgical approaches without adequately addressing the long-term impacts of surgical intervention on quality of life and symptom relief in specific patient demographics. Notably, our study highlights that patients with HH-GERD and extra-esophageal symptoms experience distinct challenges, necessitating tailored treatment protocols that consider their unique profiles. This aligns with the conclusions drawn by previous research, indicating that individualized treatment plans significantly enhance patient outcomes and satisfaction (24, 25).

The implications of our findings extend to clinical practice and policy formulation, emphasizing the need for a shift towards surgical intervention as a viable option for managing refractory GERD symptoms. The marked elevation in the EORTC QLQ-C30 functional scales, particularly in role and emotional functioning, underscores that the benefit of LHHR extends beyond mere symptom control. It significantly restores patients' capacity for daily activities and improves their psychosocial well-being. This is particularly relevant for patients who have not responded adequately to pharmacological therapies, as highlighted in contemporary literature that advocates for surgical options in cases of persistent symptoms despite medical management (26, 27). As such, healthcare providers should consider incorporating surgical interventions into treatment algorithms for patients with complex presentations of HH-GERD, ensuring that patients are informed about the potential benefits and risks associated with these procedures.

The results of this study showed that the DeMeester index in both the LNF group and the LTF group significantly decreased after surgery: in the LNF group, it decreased from 55.23 ± 25.12 to 11.45 ± 10.20, and in the LTF group, it decreased from 60.51 ± 28.40 to 11.70 ± 9.65 (*P* < 0.05). The RSI scores in both groups also significantly improved 12 months after surgery: in the LNF group, it decreased from 23.1 ± 15.4 to 13.7 ± 9.6, and in the LTF group, it decreased from 21.9 ± 15.8 to 12.8 ± 8.2 (*P* < 0.05). The GERD scores improved after surgery as well: in the LNF group, it decreased from 13 ± 5.0 to 10 ± 4.4, and in the LTF group, it decreased from 10 ± 4.7 to 7.5 ± 4.5 (*P* < 0.05). In addition, patients in both the LTF and LNF groups experienced significant improvement in both esophageal and extra-esophageal symptoms (*P* < 0.05). These results indicate that the statistically significant improvements in various assessments highlight the effectiveness of LNF and LTF in treating GERD. Both Nissen and Toupet fundoplication have shown to be equally effective in treating GERD and enhancing patients' life quality.

There are many clinical studies on the relationship between esophageal hiatal hernia and GERD, but there are not many in-depth studies comparing systematically the two surgical modalities on the improvement of preoperative and postoperative symptoms of GERD patients with esophageal hiatal hernia. In the present study, out of concern for patients' postoperative quality of life and social psychology and based on the currently known and mature surgical modalities, we used the EORTC QLQ-C30 to compare the postoperative symptomatic improvement of the patients treated with the two surgical modalities in a more detailed manner. And the follow-up period was up to 12 months, in which the patients' quality of life scores was carefully counted and analyzed, resulting in the results presented in the article. The conclusions in the article further validate the effectiveness of the surgery and provide new clinical data for the treatment of patients diagnosed with hiatal hernia and GERD.

Nevertheless, our study is not without limitations. The relatively small sample size from a single center might limit the statistical power and may affect the generalizability of our findings. The lack of a control group prevents direct comparison with non-surgical management or other interventions. Future research should address these limitations by including larger, multicenter cohorts and long-term follow-up assessments to better understand the recurrence rates and sustained quality of life improvements post-surgery. We have only simple emotional and social assessments in our data. The absence of extensive psychological and social evaluations in our study indicates a need for comprehensive patient assessments in future research to capture the full spectrum of factors influencing treatment outcomes (28). By addressing these gaps, subsequent investigations can contribute to a more robust understanding of the multifaceted nature of HH and GERD management, ultimately enhancing patient care and outcomes. The GERD-Q questionnaire, while validated for GERD diagnosis and symptom burden assessment, may not fully capture postoperative symptom changes following fundoplication. Future studies may benefit from incorporating procedure-specific patient-reported outcome measures to better evaluate postoperative recovery.

## 6 Conclusion

This study demonstrates that patients with HH-GERD who received LHHR treatment have made significant progress in terms of symptom relief and quality of life. The statistically significant improvements in various assessments highlight the effectiveness of LNF and LTF in treating GERD. Both Nissen fundoplication and Toupet fundoplication have demonstrated comparably excellent outcomes in alleviating esophageal and extra-esophageal symptoms of GERD and in improving patients' quality of life.

## Data Availability

The original contributions presented in the study are included in the article/Supplementary Material, further inquiries can be directed to the corresponding author.
