# Supplementary material for: Comparison of Nissen vs. Toupet fundoplication in laparoscopic hiatal hernia repair for gastroesophageal reflux disease with extra-esophageal symptoms
Source: Front Med Technol. 2026 Jan 9;7:1678192. doi: 10.3389/fmedt.2025.1678192 (PMC12827679; doi:10.3389/fmedt.2025.1678192)
Supplement: Supplementary file 1 [file Datasheet1.pdf]

Supplementary Figure

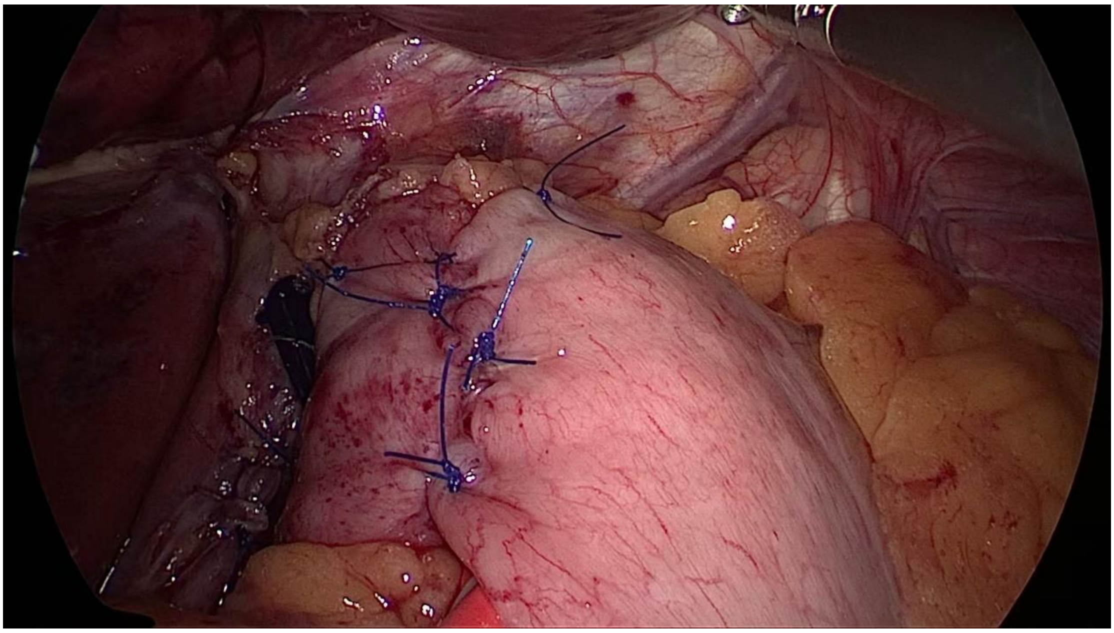

Supplementary Figure S1 Schematic Diagram of a 360-Degree Nissen Fundoplication

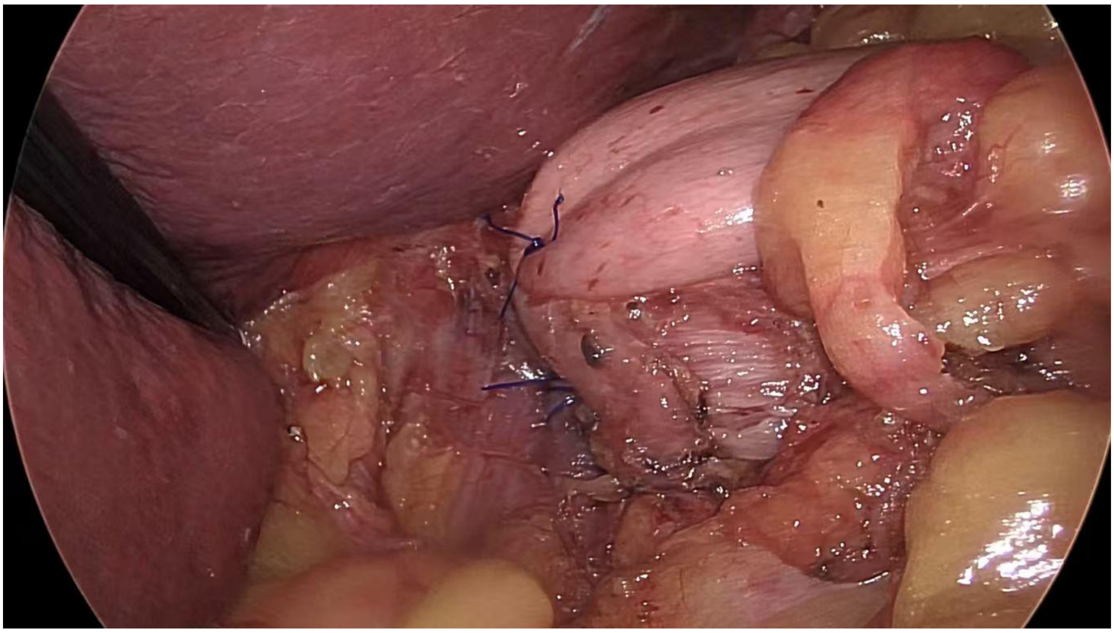

Supplementary Figure S2 Schematic Diagram of a 270-Degree Toupet Fundoplication
